# Soil Microbial Composition and *phoD* Gene Abundance Are Sensitive to Phosphorus Level in a Long-Term Wheat-Maize Crop System

**DOI:** 10.3389/fmicb.2020.605955

**Published:** 2021-01-14

**Authors:** Ming Lang, Wenxin Zou, Xiuxiu Chen, Chunqin Zou, Wei Zhang, Yan Deng, Feng Zhu, Peng Yu, Xinping Chen

**Affiliations:** ^1^College of Resources and Environment, Academy of Agricultural Sciences, Southwest University, Chongqing, China; ^2^Interdisciplinary Research Center for Agriculture Green Development in Yangtze River Basin, Southwest University, Chongqing, China; ^3^Center for Resources, Environment and Food Security, College of Resources and Environmental Sciences, China Agricultural University, Beijing, China; ^4^Chongqing Key Laboratory of Efficient Utilization of Soil and Fertilizer Resources, Southwest University, Chongqing, China; ^5^Key Laboratory of Agricultural Water Resources, Hebei Key Laboratory of Soil Ecology, Center for Agricultural Resources Research, Institute of Genetic and Developmental Biology, Chinese Academy of Sciences, Shijiazhuang, China; ^6^Crop Functional Genomics, Institute of Crop Science and Resource Conservation (INRES), University of Bonn, Bonn, Germany

**Keywords:** phosphorus forms, bacterial and fungal communities, keystone taxa, microbial network analysis, *phoD* gene

## Abstract

Microbes associated with phosphorus (P) cycling are intrinsic to soil P transformation and availability for plant use but are also influenced by the application of P fertilizer. Nevertheless, the variability in soil P in the field means that integrative analyses of soil P cycling, microbial composition, and microbial functional genes related to P cycling remain very challenging. In the present study in the North China Plain, we subjected the bacterial and fungal communities to amplicon sequencing analysis and characterized the alkaline phosphatase *gene* (*phoD)* encoding bacterial alkaline phosphatase in a long-term field experiment (10 years) with six mineral P fertilization rates up to 200 kg P ha^–1^. Long-term P fertilization increased soil available P, inorganic P, and total P, while soil organic P increased until the applied P rate reached 25 kg ha^–1^ and then decreased. The fungal alpha-diversity decreased as P rate increased, while there were no significant effects on bacterial alpha-diversity. Community compositions of bacteria and fungi were significantly affected by P rates at order and family levels. The number of keystone taxa decreased from 10 to 3 OTUs under increasing P rates from 0 to 200 kg ha^–1^. The gene copy numbers of the biomarker of the alkaline phosphatase *phoD* was higher at moderate P rates (25 and 50 kg ha^–1^) than at low (0 and 12.5 kg ha^–1^) and high (100 and 200 kg ha^–1^) rates of P fertilization, and was positively correlated with soil organic P concentration. One of the keystone taxa named BacOTU3771 belonging to Xanthomonadales was positively correlated with potential functional genes encoding enzymes such as glycerophosphoryl diester phosphodiesterase, acid phosphatase and negatively correlated with guinoprotein glucose dehydrogenase. Altogether, the results show the systematic effect of P gradient fertilization on P forms, the microbial community structure, keystone taxa, and functional genes associated with P cycling and highlight the potential of moderate rates of P fertilization to maintain microbial community composition, specific taxa, and levels of functional genes to achieve and sustain soil health.

## Introduction

Continuous application of phosphorus (P) fertilizer to achieve high productivity in intensive agro-ecosystems is known to cause high P accumulation in soil (MacDonald et al., 2011; Zhu et al., 2018) and environmental risk (MacDonald et al., 2011; Peñuelas et al., 2013; Withers et al., 2014; Zhang L. et al., 2018). Microorganisms are integral to soil P cycling (Khan et al., 2014) and play an important role in mediating the availability of P to plants (Richardson and Simpson, 2011). A comprehensive understanding of the effects of the P application on soil P cycling-related microorganisms is urgently needed in order to establish P management strategies for improving P use efficiency and maintaining soil health in sustainable-intensive agriculture. Considering the provision of P fertilizers is limited and will be exhausted in a few decades, and thus new research is required on the topic.

The soil microbiome and its functional capacity determines P availability in soils (Bargaz et al., 2018). Several studies have highlighted the variation of microbial diversity under conditions of increasing P fertilization (Chhabra et al., 2013; Liu M. et al., 2018; Ye et al., 2020). At the same time, there are contrasting findings on the structure of the microbial community in response to soil P gradients. Long-term P fertilization impacted the soil fungal and bacterial community but not arbuscular mycorrhiza fungi community (Beauregard et al., 2010); the alteration of microbial community size and activity was affected by the carbon compounds (Wakelin et al., 2017); the abundance of most groups of the soil microbial community including bacteria, fungi and arbuscular mycorrhizal fungi increased with P addition (Huang et al., 2016). However, the soil microbial community was adaptable and kept stable to a wide range of soil conditions with different nutrient input treatments for 37 years (Hamel et al., 2006). Such divergent results indicate the complexity and instability of soil microbiomes influenced by edaphic factors.

Inorganic P is the main form of mineral P fertilizer for intensive agriculture (Liu et al., 2019, 2020). It is well recognized that microbial functional genes encoding inorganic P solubilizing enzymes likepyrroloquinoline, do affect the production of organic acids such as gluconic acid, acetic acid and citric acid (Rodríguez et al., 2006; Long et al., 2018). Worldwide, organic P accounts for more than 40% on average in the agriculture soil (Menezes-Blackburn et al., 2018) and plays a key role in available P supply to plants. Bacteria harboring phosphatase genes have the capacity to mineralize organic P from soil by producing phosphatase (Fraser et al., 2015, 2017), which is often found negatively (Fraser et al., 2015, 2017) or positively (Hu et al., 2019) correlated with soil available P concentration as influenced by P fertilization (Smith and Smith, 2011; Mander et al., 2012; Ragot et al., 2016). It was found that high input of mineral P fertilizer could in turn change community composition and reduce the proportion of *phoD*-harboring bacteria (indicated by the biomarker for the alkaline phosphatase gene) (Tan et al., 2013; Fraser et al., 2015; Lagos et al., 2016). In a previous study it was showed that microbiome with *phoD*-harboring microbes had the potential to immobilize organic P when the supply was sufficient, while mineralizing organic P under P-poor conditions. However, the amount of organic P with the increased P addition is uncertain. The results showed that they can increase (Liu et al., 2017), decrease (Cade-Menun et al., 2017), or do not change (van der Bom et al., 2019) when P fertilization rates increased. In addition, most of the research has focused mainly on the relationship between available P in soil and *phoD* gene copy numbers, whereas the relationship between organic P and *phoD* abundance has not been very clear. Besides, the organic P mineralization was carried out by fungi, specially, soil fungi including *Geastrum sp.* and *Chaetomium sp.* played an important role in mineralizing organic P in acidic soil (Chen Y. et al., 2020). It is of great importance to explore the relationship between the overall microbes and the organic P.

Correlation-based network analysis is a biologically meaningful tool to predict the keystone and functional microbial taxa (Röttjers and Faust, 2018). Recently, specific compositional shifts in the keystone phyla of the bacterial community from Proteobacteria and, Actinobacteria to Firmicutes were found to correspond to levels of applied P (no P, moderate and excessive) in rice paddy soil (Long et al., 2018). In a study of the rhizosphere soil of a peanut crop, the relative abundance of two keystone bacterial operational taxonomic units (OTUs) belonging to *Chitinophaga* and *Nitrospira* positively correlated with P fertilization and displayed high potential to promote carbon-P and nitrogen-P synergistic conversion, respectively (Chen et al., 2018). Thus, P level and crop species could affect the keystone microbial taxa with specific functional roles in agriculture.

Despite these recent studies, the diversity of the soil microbiome and its contribution to soil P cycling in response to continuous mineral P fertilization in the long-term field remain largely unknown. The keystone taxa and functional genes of the soil microbiome in maize crops remain to be identified. A deeper understanding of the role of functional genes, which are relevant to P cycling (including inorganic P dissolution and organic P mineralization), is the key to exploitation use of recalcitrant P in intensive long-term P fertilization fields. In the present study we subjected the microbiome to functional gene profiling during a gradual increase of soil P accumulation over a 10-year fertilization regime. By application of high-throughput sequencing (16S and ITS), we characterized the composition and diversity of the bacterial and fungal communities. Moreover, real-time qPCR was used to quantify the expression of the functional gene *phoD* involved in P cycling in soil. We hypothesis that P application rates are the major trigger contributing to reshaping of the microbial community structure, establishment of keystone taxa and maintenance of functional genes, with the result of efficient P cycling in the long-term maize field. This discovery provides the theoretical foundation on which future studies can be based to understand the balance between plant P use efficiency and field P management strategy in sustainable-intensive agriculture.

## Materials and Methods

### Experimental Setup

The field experiment was set up at the Quzhou Experimental Station, China (36°52′N, 115°02′E), in an area of intensive agriculture in the North China Plain. The annual mean temperature is 13.2°C, and annual precipitation is 494 mm. The soil type is an alluvial loam with basic properties as follows: pH 7.3 (water: soil ratio 2: 1), 10.3 g kg^–1^ organic matter, 0.67 g kg^–1^ total nitrogen, 7.0 mg kg^–1^ available P (by Olsen method) (Olsen et al., 1982), and 74 mg kg^–1^ exchangeable potassium.

Winter wheat-summer maize rotation system was established in 2008 for the long-term P fertilization experiment. In each rotation, wheat was sown in October and harvested in June of the following year, after which maize was immediately planted for harvest in October. The experimental design was a randomized block. The P fertilization gradient was set with P input at 0, 12.5, 25, 50, 75, and 100 kg ha^–1^ P for winter wheat and 0, 6.25, 12.5, 25, 37.5, and 50 kg ha^–1^ P for summer maize from October, 2008 to July, 2009. Then the P rates were changed to 0, 25, 50, 100, 200, and 400 kg ha^–1^ P for winter wheat and with 0, 12.5, 25, 50, 100, and 200 kg ha^–1^ P for summer maize after July, 2009. We collected the soil samples in the maize season for the six P rates in the year 2018.

Different rates of P fertilization were applied in the form of calcium superphosphate, while 75 kg ha^–1^ nitrogen as urea and 50 kg ha^–1^ potash as potassium sulfate were applied before sowing, and another 150 kg ha^–1^ nitrogen as urea was top-dressed at the jointing stage for wheat and the same amount was applied at the 12-leaf stage for maize, respectively. Each treatment consisted of three replicated plots, with each plot having an area of 43.2 m^2^ (5.4 m × 8 m).

Our previous study showed that the optimal P rates for yield and P uptake were 12.5–25 kg ha^–1^ for summer maize or 25–50 kg ha^–1^ for winter wheat in single seasons (Teng et al., 2013; Deng Y. et al., 2014; Deng et al., 2017; Zhang W. et al., 2018). In the last five years, the agronomic optimum P rates almost stabilized in the range of 25–50 kg ha^–1^ for both summer maize and for winter wheat (2014–2018) (Supplementary Figure 1). Therefore, the six P rate treatments could be divided into three phases for summer maize in this study, among which P0-P12.5, P25-P50, and P100-P200 were defined as low P, moderate P, and high P, respectively.

### Sampling and Soil P Properties Analysis

Soil samples were taken at 26 days after sowing (maize V6 stage on July 4, 2018). From each plot, soil cores of 20 cm depth were taken from five different locations between plant rows and they were combined to form a single replicate sample. The combined soil sample was divided into two portions after sieving with < 2 mm mesh. One portion was stored at −20°C for DNA extraction and subsequent amplicon sequencing, and the other portion was air-dried for chemical analysis of soil P properties including Olsen P, total P, inorganic P, and organic P. Soil available P (Olsen P) was extracted with 0.5 M NaHCO_3_ at pH 8.5 and then determined by colorimetry (Olsen et al., 1982). The total P in the soils was determined by using the acid digestion method (Tiessen, 1993). Inorganic P was calculated as the total P minus organic P. Organic P was determined by the ignition method (Saunders and Williams, 1955).

### DNA Extraction, PCR Amplification and Illumina Sequencing

Soil DNA was extracted from 0.5 g frozen soil samples using a FastDNA SPIN Kit (MP Biomedicals, Santa Ana, CA, United States) following the manufacturer’s instructions. The concentration and quality of the extracted DNA samples were check spectrophotometerically (NanoDrop Technologies, Wilmington, DE, United States) and visually under a 1% (w/v) agarose gel. Illumina MiSeq high-throughput sequencing was performed to investigate the diversity and composition of the soil fungal and bacterial communities. The primers 515F (5′- GTGCCAGCMGCCGCGG -3′) and 907R (5′- CCGTCAATTCMTTTRAGTTT -3′) targeting the V4-V5 hypervariable regions of the bacterial 16S ribosomal RNA gene and the fungal specific primers 1737F (5′-CTTGGTCATTTAGAGGAAGTAA-3′) and 2043R (5′-TCCTCCGCTTATTGATATGC-3′) for the fungal ITS1 region were selected for PCR amplification. Each barcode unique sequence was added to the forward primer of each sample. PCR was carried out with 10 ng template DNA, 0.8 μl of each primer (both at 5 μM), 4 μl 5 × FastPfu Buffer, 2 μl 2.5 mM dNTPs, and 0.4 μl FastPfu polymerase (TransGen Biotech, Beijing, China) and mixed together to a final volume of 20 μl with distilled water. The thermal conditions for the bacterial 16S rRNA gene and fungal ITS region were 95°C for 3 min, 27 cycles at 95°C for 30 s (33 cycles for ITS), 55°C for 30 s and 72°C for 45 s of extension, followed by 72°C for 10 min. PCR products were then purified and mixed in equimolar ratios to obtain a quantitative sample DNA library that was further used for sequencing. Raw sequences were demultiplexed and quality-filtered using the Quantitative Insights Into Microbial Ecology (QIIME) toolkit (version 1.19). The primers were removed and sequenced with a quality score < 20 or with any truncated reads shorter than 50 bp were eliminated for further analysis. OTUswere then clustered at the 97% sequence similarity level using the Usearch program which provided clustering, chimera checking, and quality filtering in QIIME. Finally, the most abundant sequence for each OTU was selected as the representative OTU and taxonomic annotations were assigned to each OTU’s representative sequence against the SILVA (SSU117/119) 16S rRNA database for bacteria and UNITE fungal ITS. Because the sequencing depth varied across samples, we used a sub-sampling procedure to normalize the number of reads to the minimum observed across all samples (30319 and 21099 reads in bacterial and fungal data, respectively). Finally, the raw sequencing data were deposited in the National Center for Biotechnology Information database under the accession number PRJNA559597 (bacteria) and PRJNA559609 (fungi).

### Quantitative PCR

The *phoD* gene was amplified with primers ALPS-F730 (5′-CAGTGGGACGACCACGAGGT-3′) and ALPS-1101 (5′-GAGGCCGATCGGC-ATGTCG-3′) (Sakurai et al., 2008; Lagos et al., 2016), and the size of amplicon is 371 bp. Each sample involved three technical replicates for the *phoD* gene amplification, which was carried out in an ABI 7500 Cycle Real-time PCR System (Applied Biosystems, Germany). 25 μl of solution contained 12.5 μl of SYBR^®^ Premix Ex Taq (2 × RNase Plus), 0.5 μl of ROX Reference Dye II (50 × ; TAKARA, BIO, Inc., Japan), 0.5 μl of each primer, 1 μl of template and ddH_2_O added to bring to a volume. Cycling conditions were as follows: 95°C for 30 s, followed by 40 cycles at 95°C for 5 s and at 60°C for 34 s. The plasmid for the standard curve was constructed according to Fraser et al. (2015, 2017). The standard curve was prepared using serial 10-fold dilutions, and the number of gene copies was calculated by measuring the concentration of the plasmid and the number of base pairs. Amplification efficiencies ranged from 96 to 98% with *R*^2^-values of 0.9992.

### Enrichment and Network Analyses

Heatmaps were plotted using HemI toolkit (Deng W. K. et al., 2014) to investigate the effects of gradient P rates on the relative abundance of bacteria and fungi at the family level.

To determine the complex ecological interactions among bacterial and fungal OTUs under different P fertilization rates, co-occurrence networks were created using the random matrix theory (RMT)-based approach to delineate phylogenetic molecular ecological networks from the high-throughput sequencing data according to the Molecular Ecological Network Analyses Pipeline (http://ieg4.rccc.ou.edu/mena/) (Zhou et al., 2010, 2011; Deng et al., 2012). To reduce rare OTUs in the data set, we removed OTUs with average relative abundances of less than 0.01% (Ma et al., 2016) of the total number of bacterial and fungal sequences. To visualize the association in the network, the co-occurrence network was inferred based on the Spearman correlation matrix (*P* < 0.01). To explore the interrelationships of bacterial and fungal OTUs, the topological characteristics of the networks were calculated as follows: average clustering coefficient, average connectivity, average path distance, and modularity. Furthermore, the topological roles of individual network nodes were determined based on two properties, including plots of Zi (measuring how well a node was connected to other nodes within its module) and Pi (measuring how well a node was connected to nodes between different modules), which have been proposed to represent keystone taxa that have potentially strong interactions with the target microbial community by modulating the bacteria-fungi relationships (Deng et al., 2012). Based on the co-occurrence networks, the keystone taxa that play key roles in the overall networks were defined as “module hubs” (highly connected to numerous OTUs in their own modules, Pi > 6.2), and “connectors” (highly linked to some modules, Zi > 2.5) (Guimera and Nunes, 2005; Liang et al., 2016; Jiang et al., 2017).

### Functional Prediction of Soil Microbial Communities

We assigned functional profiles for the six P treatments (P0, P12.5, P25, P50, P100, and P200) using the PICRUSt2 software package (Douglas et al., 2020). This technique estimates genomic abundance of potential functional genes by KEGG orthology (KO) in an OTU from an environmental sample based on the position of those OTUs in a reference phylogeny of complete microbial genomes. Functional-profile assignments were made based on partial 16S rRNA gene sequences, and the sequences were mapped to the Greengenes 13_8 reference phylogeny using QIIME (Morrow et al., 2015).

### Statistical Method

Significant differences in soil physicochemical properties, P forms, microbial abundance and alpha-diversity data among fertilization treatments were examined using one-way analysis of variance and mean values were compared with Duncan’s multiple range test at the 5% level using the SPSS version 20.0 software package (SPSS, Inc., Chicago, IL, United States). Estimates of alpha-diversity were based on OTU abundance matrices and included OTU number and Shannon’s index calculated in the R Vegan package (Hammer et al., 2001). A principal coordinate analysis (PCoAs) on the OTU level based on the Bray-Curtis distance metric was used to compare the beta-diversity between treatments of bacterial and fungal communities. Community structures of bacteria and fungi for different P fertilization treatments were investigated according to permutational multivariate analysis of variance (PERMANOVA) by R Vegan package with 999 permutations (Anderson, 2001). This was performed with the R software package (version 3.3.1) using the ‘ape’ library (Paradis et al., 2004). Redundancy analysis (RDA) was performed to investigate the relationships between environmental variables and soil samples of each treatment at the OTU level of bacteria and fungi using the function ‘envfit’ in Vegan (Oksanen et al., 2013). Pearson’s correlation was performed to estimate the relationships of bacterial and fungal communities at class and family levels and environmental variables, including Olsen P, inorganic P, organic P and total P; the relationships between the number of *phoD* copies and organic P concentration; the relationship between potential functioning genes and the relative abundance of the keystone taxa. A linear discriminant analysis effect size (LEfSe) was performed to identify significant differences in bacterial and fungal taxa among fertilization treatments using the non-parametric factorial Kruskal-Wallis (KW) sumrank test and then the effect size of each differentially abundant feature was estimated with linear discriminant analysis (Segata and Blanzieri, 2011). We performed statistical analysis of LEfSe of bacterial and fungal communities from kingdom to genus levels, and the threshold LDA score ≥ 3.0 (Bei et al., 2018) was considered as signifying important contributors to the model.

## Results

### Variations of Soil P Forms in the Long-Term Field

**FIGURE 1:**
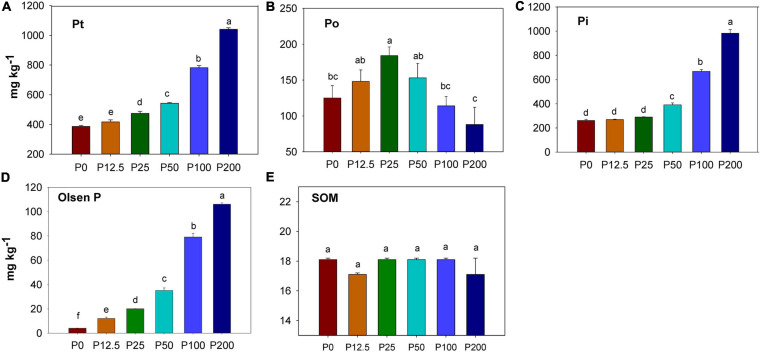
Concentration of soil total P (Pt) **(A)**, organic P (Po) **(B)**, inorganic P (Pi) **(C)**,Olsen P **(D)** and soil organic carbon (SOM) **(E)** depending on P fertilization. Abbreviations: P0, P12.5, P25, P50, P100, and P200 represent 0, 12.5, 25, 50, 100, and 200 kg P ha^–1^, respectively. The units of all Y axes are mg kg^–1^. Different lower case letters denote significantly different means on *P* < 0.05.

Total P and inorganic P increased as P fertilization rate increased (Figures 1A,C). Soil organic P firstly increased as P fertilization increased, but then decreased when P rates were higher than 25 kg ha^–1^ (Figure 1B). A significant difference in soil available P (by Olsen method) was found among treatments after ten years’ (20 crop seasons) of continuous application of inorganic P fertilizer. Compared with P0 treatment (4.2 mg kg^–1^), soil available P was 12.3 mg kg^–1^ in the P12.5 treatment, 20.1 mg kg^–1^ and 35.7 mg kg^–1^ in the P25 and P50 treatments, and 79.6 mg kg^–1^ and 106.2 mg kg^–1^ in the P100 and P200 treatments (Figure 1D). The soil organic matter (SOM) had no significant difference among different P fertilization treatments (Figure 1E).

### Alpha- and Beta-Diversity of Bacterial and Fungal Communities

**FIGURE 2:**
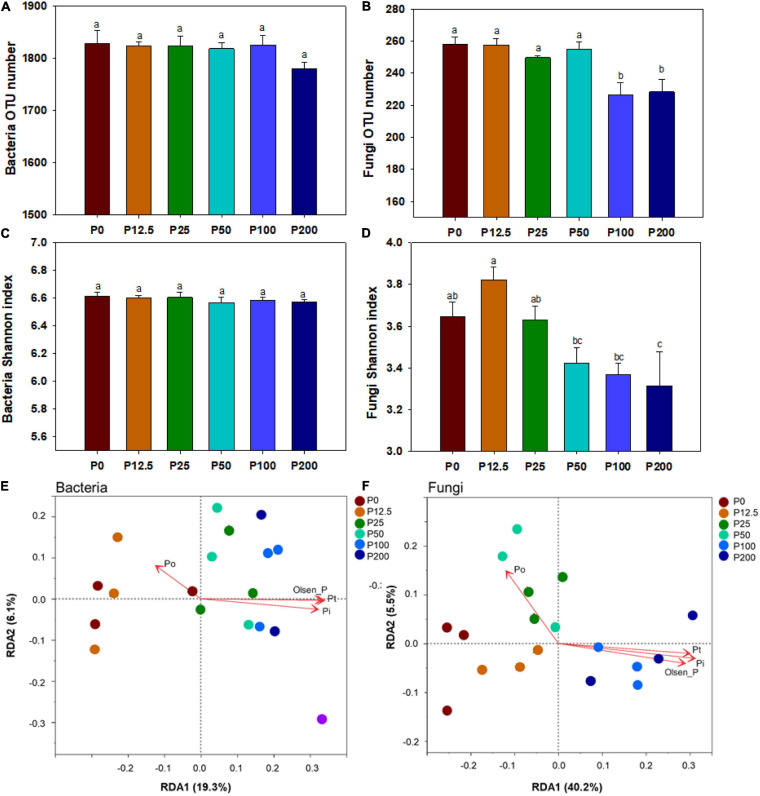
The bacterial OTU number **(A)**, fungal OTU number **(B)**, bacterial Shannon index **(C)**, fungal Shannon index **(D)**; Different lower case letters denote significantly different means on *P* < 0.05. Redundancy analysis (RDA) of soil physicochemical properties of bacteria **(E)** and fungi **(F)** under different P fertilization treatments. Soil physicochemical properties are fitted as vectors onto each ordination plot. Abbreviations: Po, organic phosphorus. Dotted circles with different color represent the distance between the microbial communities obtained from corresponding P fertilization rates. Abbreviations: P0, P12.5, P25, P50, P100, and P200 represent 0, 12.5, 25, 50, 100, and 200 kg P ha^–1^, respectively.

The alpha-diversity was estimated by OTU number and Shannon’s index. There were no significant differences in bacterial OTU number and Shannon’s index among all six treatments (*P* > 0.05) (Figures 2A,C). In contrast, the fungal OTU number decreased significantly when P rates exceeded 50 kg ha^–1^ (Figure 2B). Especially, the fungal Shannon’s index gradually decreased with P application from P12.5 to P200 (Figure 2D).

### Structure and Composition of Bacterial and Fungal Communities

High-throughput sequencing revealed that 11 dominant phyla of bacteria accounted for 94-96% of the total bacterial sequences (Supplementary Figure 2a), while three fungal phyla, Ascomycota, Zygomycota and Basidiomycota accounted for 98-99% of the total fungal sequences (Supplementary Figure 2b). At the phylum level, only Acidobacteria and Bacteroidetes bacteria were affected by P fertilization. At the class level, only the proportion of Actinobacteria increased significantly as P rates increased, and the proportion of Tremellomycetes fungi decreased significantly as P rates increased and this decrease was significantly correlated with Olsen P, inorganic P and total P (Supplementary Tables 1, 2).

**FIGURE 3:**
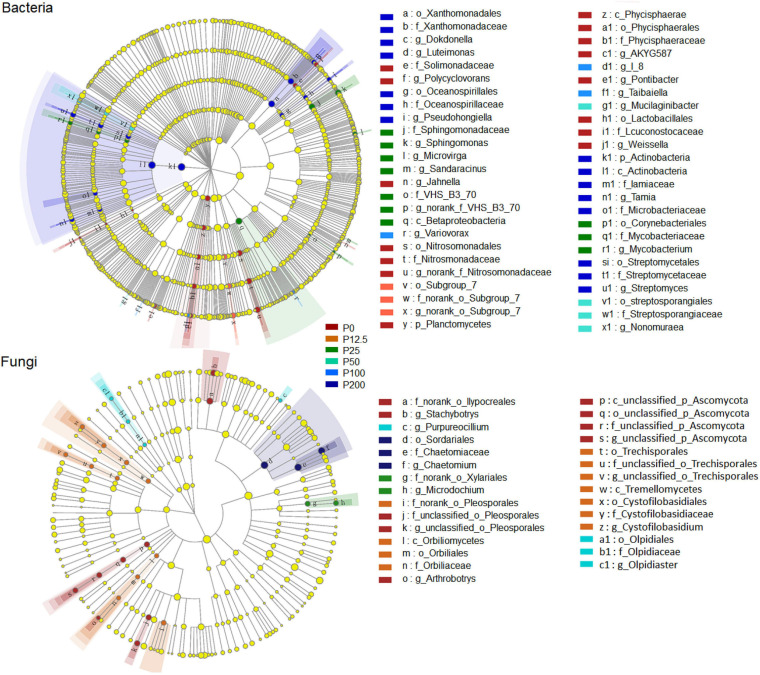
A linear discriminant analysis effect size (LEfSe) method identifies the significantly different abundant taxa of bacteria and fungi with linear discriminant analysis (LDA) score higher than 3.0. Circles represent phylogenetic levels from kingdom to genus. The taxa with significantly different abundances among P0, P12.5, P25, P50, P100, and P200 P fertilization levels are represented by colored dots. Abbreviations: P0, P12.5, P25, P50, P100, and P200 represent 0, 12.5, 25, 50, 100, and 200 kg P ha^–1^, respectively.

**FIGURE 4:**
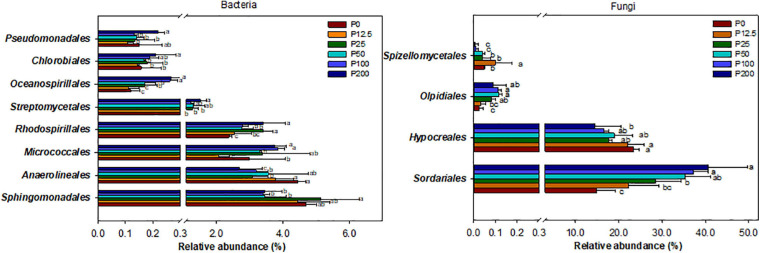
Relative abundance of selected bacterial (left) and fungal (right) orders for different long-term P fertilization treatments of the maize field. The one-way analysis of variance was used to test for significant differences between different treatment; only taxa with relative abundance > 0.1% in at least one sample were included in the analysis, and only order with significant (*P* < 0.05) response to the treatments were presented. Abbreviations: P0, P12.5, P25, P50, P100, and P200 represent 0, 12.5, 25, 50, 100, and 200 kg P ha^–1^, respectively.

The community structures of both bacteria and fungi were significantly affected by P forms including total P, inorganic P, and Olsen P (Figures 2E,F and Supplementary Table 3). LEfSe analysis showed that bacteria and fungi strongly shifted in relative abundance at the order level for P fertilization (Figure 3). At the order level, bacterial relative abundances of Rhodospirillales, Streptomycetales, Oceanospirillales, Chlorobiales, and Pseudomonadales and fungal relative abundance of Sordariales and Olpidiales increased with P rates increased; while bacterial relative abundances of Anaerolineales and fungal relative abundance of Hypocreales decreased with P rates increased. The bacterial relative abundances of Sphingomonadales and fungal relative abundance of Spizelomycetales at first increased and then decreased when the P rates were higher than 50 kg ha^–1^ and 25 kg ha^–1^, respectively (Figures 4A,B). We used heatmaps to illustrate relative enrichments in the bacterial and fungal compositions among P rate treatments at the family level (Supplementary Figure 3). The detailed results were described in Supplementary Results.

### Keystone Taxa

Using co-occurrence network analysis, we identified clearly different structures among the low P (P0-P12.5), moderate P (P25-P50) and high P (P100-P200) treatments (Supplementary Figure 4). The total nodes and edges in the network module decreased from low P (P0-P12.5) to moderate P (P25-P50), while the total nodes and edges increased from moderate P (P25-P50) to high P (P100-P2100) (Table 1). The ratio of the positive to total edges increased with P application rates (Supplementary Figure 4). The average connectivity and average clustering coefficient increased as P rates increased (Table 1).

**TABLE 1:** Topological properties of the empirical phylogenetic molecular ecological networks (pMENs) of bacterial and fungal communities in low P, moderate P and high P maize field soil and their associated random pMENs.

	Empirical networks											Random networks		

Treatments	Node	Edge	Positive edges	Negative edges	No. of original OTUs	Similarity threshold	R square of power-low	Average degree	Average clustering coefficient	Average path distance	Modularity	Average path distance ± SD	Average clustering coefficient ± SD	Average modularity ± SD
Low P	238	581	249	332	1960	0.91	0.574	4.432	0.303	6.193	20	3.910 ± 0.031	0.017 ± 0.006	0.466 ± 0.008
Moderate P	101	364	204	160	1960	0.93	0.887	4.460	0.331	6.034	22	3.646 ± 0.053	0.331 ± 0.008	0.448 ± 0.008
High P	169	648	270	378	1960	0.92	0.742	5.878	0.363	6.351	20	3.266 ± 0.038	0.054 ± 0.008	0.367 ± 0.006

**FIGURE 5:**
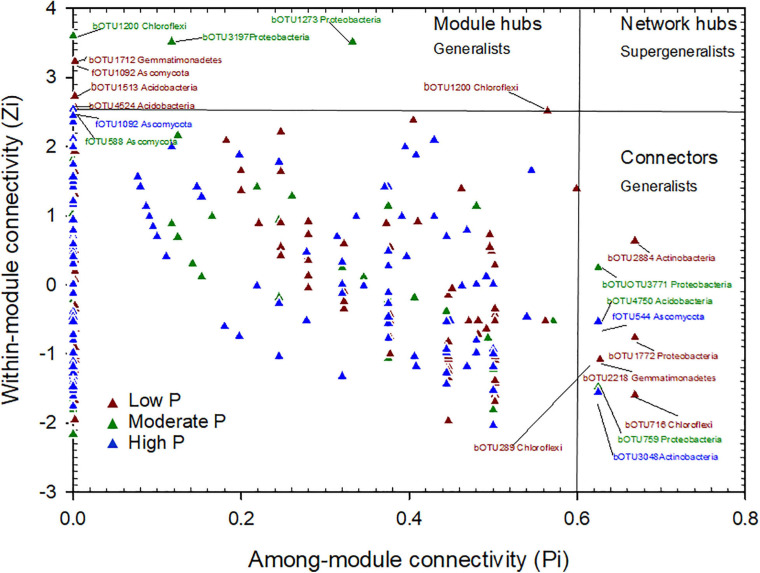
Zi-Pi plot showing the distribution of OTUs based on their topological roles. Each symbol represents an OTU in the low P (red triangle), moderate P (green triangle) and high P (blue triangle) network of maize field. The threshold values of Zi and Pi for categorizing OTUs were 2.5 and 0.62, respectively.

The plot of Zi (within-module connectivity) and -Pi (among-module connectivity) illustrates the topological roles of individual network nodes in the P fertilization treatments (Figure 5). Both the module hub and connectors were defined as keystone taxa. There were 5 OTUs as module hubs and 5 OTUs as connectors in the low P network. The module hubs belonged to the phyla of Actinobacteria, Proteobacteria, Chloroflexi, Gemmatimonadetes, and connectors belonged to the phyla of Gemmatimonadetes, Acidobacteria, Chloroflexi and Ascomycota. In the moderate P network, 4 OTUs were module hubs and belonged to the phyla Proteobacteria, Chloroflexi and Ascomycota, while the connectors were 3 OTUs of the phyla Proteobacteria and Acidobacteria. In the high P network, one OUT, belonging to the phylum Ascomycota, 2 OTUs (in the phyla Actinobacteria and Ascomycota) were connectors (Figure 5 and Table 2). Thus, the number of keystone taxa (both as connectors and module hubs) in networks decreased gradually as P rates increased (Table 1).

**TABLE 2:** Information of the nodes identified as module hubs or connectors of bacterial networks among the low P, moderate P and high P. Low P, moderate P and high P include 0–12.5 kg P ha^–1^, 25–50 kg P ha^–1^ and 100–200 kg P ha^–1^, respectively.

Treatment	ID	Generalists	Phylum	Class	Order	Family	Genus
Low P	BacOTU2884	Connnector	Actinobacteria	Actinobacteria	Gaiellales		
	BacOTU1772	Connnector	Proteobacteria	Alphaproteobacteria	Rhodospirillales	*Rhodospirillaceae*	
	BacOTU716	Connnector	Chloroflexi	Anaerolineae	Anaerolineales	*Anaerolineaceae*	
	BacOTU2218	Connnector	Gemmatimonadetes	Gemmatimonadetes			
	BacOTU289	Connnector	Chloroflexi	Anaerolineae	Anaerolineales	*Anaerolineaceae*	
	BacOTU1712	Module hubs	Gemmatimonadetes	Gemmatimonadetes	Gemmatimonadales	*Gemmatimonadaceae*	
	BacOTU1513	Module hubs	Acidobacteria	Acidobacteria			
	BacOTU4524	Module hubs	Acidobacteria	Acidobacteria	Solibacterales	*Solibacteraceae*	*PAUC26f*
	BacOTU1200	Module hubs	Chloroflexi	Chloroflexia	Chloroflexales	*Roseiflexaceae*	*Roseiflexus*
	FunOTU1092	Module hubs	Ascomycota	Sordariomycetes	Hypocreales	*Nectriaceae*	
Moderate P	BacOTU3771	Connnector	Proteobacteria	Gammaproteobacteria	Xanthomonadales		
	BacOTU759	Connnector	Proteobacteria	Alphaproteobacteria	Rhizobiales	*Hyphomicrobiaceae*	*Rhodomicrobium*
	BacOTU1200	Module hubs	Chloroflexi	Chloroflexia	Chloroflexales	*Roseiflexaceae*	*Roseiflexus*
	BacOTU1273	Module hubs	Proteobacteria	Betaproteobacteria	Nitrosomonadales	*Nitrosomonadaceae*	
	BacOTU3197	Module hubs	Proteobacteria	Betaproteobacteria	Nitrosomonadales	*Nitrosomonadaceae*	
	FunOTU588	Module hubs	Ascomycota	Sordariomycetes	Sordariales	*Chaetomiaceae*	*Chaetomium*
High P	BacOTU3048	Connnector	Actinobacteria	Actinobacteria	Corynebacteriales	*Mycobacteriaceae*	*Mycobacterium*
	FunOTU544	Connnector	Ascomycota	Sordariomycetes	Hypocreales	*Nectriaceae*	*Fusarium*
	FunOTU1092	Module hubs	Ascomycota	Sordariomycetes	Hypocreales	*Nectriaceae*	

### Microbial P Cycling Functional Gene and Its Relationship With Soil P Forms and the Keystone Taxa

**FIGURE 6:**
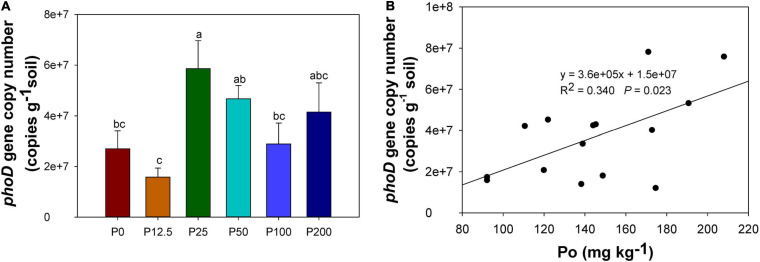
*phoD* gene copy numbers in soil **(A)**, correlation between *phoD* gene copy number and organic P concentration **(B)**. Abbreviations: P0, P12.5, P25, P50, P100, and P200 represent 0, 12.5, 25, 50, 100, and 200 kg P ha^–1^, respectively. Different lower case letters denote significantly different means on *P* < 0.05.

Alkaline phosphatase encoded by the bacterial harboring *phoD* gene could promote the organic P mineralization. The copy numbers of *phoD* gene were the highest when the P rate increased to 25 kg ha^–1^, and then decreased when P rates further increased (Figure 6A). The number of copies of *phoD* were significantly correlated with soil organic P concentration (Figure 6B) but not with the concentration of other P forms.

To gain further insights into the potential functional genes of the microbiome, 9 genes relevant to P mineralization and solubilization were evaluated (Supplementary Table 5). Among them, potential functional genes encoding enzymes named glycerophosphoryl diester phosphodiesterase and acid phosphatase were higher in the P200 treatment compared with other P treatments. Potential functional genes encoding enzymes named guinoprotein glucose dehydrogenase decreased with the increased of P fertilization; there were no significant difference among P fertilization treatments of other P cycling genes (Supplementary Table 5). Meanwhile, the relationship between the relative abundance of the keystone taxa and their associated functional structure was also analyzed, the results showed that some of the keystone taxa were significantly correlated with potential functioning genes. For example, one of the keystone taxa named BacOTU3771 belonging to Xanthomonadales was positively correlated with potential functional genes encoding enzymes such as glycerophosphoryl diester phosphodiesterase, acid phosphatase and positively correlated with guinoprotein glucose dehydrogenase (Supplementary Table 6).

## Discussion

### Overall Diversity and Community Composition of Bacteria and Fungi in Soil With Distinct P Application Rates

In this study, the bacterial alpha-diversity was not affected by long-term P fertilization, whereas the fungal alpha diversity significantly decreased with the increase in P fertilization rates (Figures 2A–D). Meta-analysis of 60 global measurements in literature showed that fungal alpha-diversity decreased with the increased of fertilizer, which was occurred mainly in soil with pH > 6 (Ye et al., 2020), which was consistent with our results. However, microbiome exhibit functional redundancy (Banerjee et al., 2016), and bacteria had more species than that of fungi, which may existed greater functional redundancy and might be more resistant with the increase of the P fertilization. These divergent responses of bacteria and fungi to P application rates might indicate that fungi are more sensitive to soil fertility and P addition than are bacteria (He et al., 2008; Li et al., 2015). Fungal hyphae usually establish complex networks in the soil and increase the surface area available for nutrient absorption, suggesting a superior acquisition capacity for available soil P, including immobile P (Smith and Smith, 2011). Therefore, soil fungi would tend to react more sensitively than bacteria in response to P addition.

The beta-diversity of both bacteria and fungi, as determined by principal coordinate analysis shows significant variation depending on P rate (Figure 2). A global-scale study reported that the community structure of bacteria and fungi in grasslands was substantially altered in long-term P fertilization from 0 kg ha^–1^ to 100 kg P ha^–1^ (Leff et al., 2015). Other researchers also concluded that both the active bacterial and fungal community structures demonstrated significant shifts in response to short-term (< 1 year) P fertilization (ranging from 0 kg ha^–1^ to 175 kg P ha^–1^) (Silva and Nahas, 2002; Girvan et al., 2004). In contrast, no apparent changes in microbial community structure was found by short-term P fertilization (ranged from 0 kg ha^–1^ to 150 kg P ha^–1^) in some studies (Hamel et al., 2006; Liu et al., 2012). The main reason why no significant difference in microbial community structure was found in the later studies might be that the P supply level was not large enough or the fertilization duration not long enough, which might lead to relatively small differences in available P in the soil. In the current study, the large gradient of soil available P ranged from 4.2 mg kg^–1^ to 106.2 mg kg^–1^ after 10 years of consecutive P application; thus, exploring the shift in community structure at different P rate treatments.

Overall, the composition and relative abundance of bacteria and fungi at the phylum and class levels were not strongly affected by long-term P application, demonstrating that the P fertilization had no significant effect on the relatively higher taxonomic levels of bacteria and fungi. However, the composition and relative abundance of bacteria and fungi were strongly shifted at the order and family levels (Figure 4; Supplementary Figure 3). Specifically, the most abundant bacterial order Sphingomonadales, among the alpha-proteobacteria, which the relative abundance were the highest in moderate P (P25) treatment. This order reported as a growth-promote bacteria and could dissolve insoluble P (Richardson et al., 2009), demonstrating that they may play a key role in the P transformation; the bacterial orders of Streptomycetales and Pseudomonadales were the highest for high P (P200) treatments, which were similar with previous study (Chen D. et al., 2020). The fungal order Sordariales was the most abundant order, and its relative abundance increased as P rate increased, which is consistent with previous results (Ye et al., 2020). The fungal order Hypocreales was one of the dominant taxa at the order level and was enriched with low P treatment. Previously, researchers have shown that Hypocreales is notable for its ability to obtain nutrients from diverse sources under nutrient-deficient conditions (Sun et al., 2016; Kepler et al., 2017). However, studies of functional bacterial groups associated with P gradient rate at relatively low levels were not well defined and further studies are needed. These results demonstrated that under different fertilization treatments, the composition of microbial communities was sensitively and strongly interacted with each other at a relative low level of microbial taxonomy.

### Effects of P Gradient Fertilization on Microbial Keystone Taxa

Microbial communities harbor keystone taxa, which drive community composition and function irrespective of their abundance (Banerjee et al., 2018). In the present study, the keystone taxa named BacOTU3771 belonging to Xanthomonadales positively correlated with potential functional genes encoding enzymes named glycerophosphoryl diester phosphodiesterase, acid phosphatase (related to organic P mineralization) and negatively correlated with potential functional genes encoding enzymes named guinoprotein glucose dehydrogenase (related to inorganic dissolution) (Supplementary Table 6), which demonstrated that the keystone taxa were closely related to the P cycling. Proteobacteria was the main keystone taxa and acted as both module hubs and connectors in the moderate P treatment (Table 2), which was generally considered to consist of copiotrophic groups with fast growth rates which are favored by nutrient rich conditions (Pianka, 1970; Francioli et al., 2016; Fierer et al., 2007). However, the number of keystone taxa decreased as P fertilization increased (Figure 5 and Table 2), indicating that network structure may tend to disorder when P fertilization input reaches an excess level. These findings, for the first time in maize, identify differences in the taxa and number of key microorganisms that perform different functions under different P fertilization conditions.

All indices commonly used in the network such as average degree, average clustering coefficient, and average path distance were greater for each treatment than their respective random networks (Table 1). The number of nodes and edges decreased from low P (0 - 12.5 kg P ha^–1^) to moderate P (25 – 50 kg P ha^–1^) and then increased from moderate P to high P (100 – 200 kg P ha^–1^); however, the positive proportion increased and then decreased as P fertilization increased (Supplementary Figure 4). Long et al. (2018) showed that the positive edges decreased as P fertilization increased (from 0 mg P_2_O_5_ kg^–1^ to 200 g P_2_O_5_ kg^–1^), as the available P in the pot experiment ranged from 5.2 to 17.8 mg kg^–1^. The range of soil available P gradient variation was somewhat small compared to our experiment from 4.2 mg kg^–1^ to 106 mg kg^–1^. The gradient of available P caused by long-term application of P fertilizer in our study was large enough to show characteristics of microbial network comprehensively. Furthermore, our results indicated that within microbial interactions in moderate P were enhanced compared with those under P deficiency or excess, even though microbial webs were more complex at the low and high supply level.

### Effects of Soil P Forms and the Relationship With P Cycling Gene Across P Fertilization Gradient

As expected, the soil Olsen P, inorganic P and total P concentrations increased during the long-term application of inorganic P fertilizer (Figures 1A,C,D), which is consistent with the results of previous studies (Li et al., 2011; Stutter et al., 2015). Our results showed that soil organic P at first increased as P fertilization increased, but then decreased when P rates were more than 25 kg ha^–1^ (Figure 1B). One of the reasons could be that the roots were retained for ten consecutive year’s maize -wheat rotation, which may result in the organic P accumulation because of crop root residue effect (Lambers, 2006; Richardson and Simpson, 2011; Liu et al., 2017). In this experiment, root biomass was firstly promoted as P fertilizer increased and then depressed as P fertilizer was applied in excess (Deng et al., 2017). Soil organic P originated predominantly from crop input (root residues) and microbial immobilization (Condron et al., 2005). Another explanation can be the synergistic effect of higher root system or higher microbial activity under optimal P addition. The maize and wheat residues in soil for over 10 years may have resulted in the organic P being higher in the optimal P treatment (around P25-P50 in this experiment).

As the previous study reported that ALP primer used in this study seem to be biased toward alpha-proteoteobacteira *phoD* over other microbial lineages (Tan et al., 2013; Ragot et al., 2015). However, a significant positive correlation between *phoD* gene abundance and ALP activity was observed, suggesting that the identified *phoD* species might represent the majority *phoD* populations in our soil. In future studies, newly designed primers should be used to detect higher *phoD* gene diversity (Ragot et al., 2015). *PhoD* gene copy numbers were the highest for P25 treatment, as the ALP primer set were known to be bias against alphaproteobacteria (Tan et al., 2013; Ragot et al., 2015), while the relative abundance of alphaproteobacteria had no significant difference among P treatments in our study, demonstrating that the functional genes were more sensitive for the relative abundance of the community composition. Results showed that the number of copies of the *phoD* gene significantly correlated with soil organic P, and was the highest at the moderate P level (P25-P50) (Figure 6B), indicating that functional gene copy numbers can be promoted for optimal P treatments. Luo et al. (2017) showed that the alkaline phosphatase activity and *phoD* gene copy numbers declined as fertilization increased, *and phoD* gene copy numbers were significantly positively correlated with labile organic P, moderately labile organic P pool and more resistant organic P pool in a maize-wheat rotation system in a lime concretion black soil, which is consistent with our results. Moreover, Fraser et al. (2015) revealed that alkaline phosphatase activity increased while *phoD* gene copy numbers generally decrease under high P fertilization rates, and NaHCO_3_-extractable organic P of the soil had a significantly negative relationship with *phoD* gene copy numbers, which is also inconsistent with our results. One possible reason may be that the high level of speciation of organic P in the soil was significantly affected by different P fertilization rates (Liu et al., 2017) and other factors such as soil type, plant species, which resulted in the uncertain relationship between organic P and *phoD* gene copy numbers.

Our results also showed that there were no bacterial taxa significantly correlated with soil organic P at the family level, while the relative abundance of fungal family *Cucurbitariaceae* was enriched in moderate P and significantly correlated with soil organic P (Supplementary Figure 3), which demonstrated that fungi could contribute to P mineralization, being the first time this has been reported under a long-term maize field experiment. Previous work revealed that the fungi including *Geastrum sp.* and *Chaetomium sp.* had the capacity for mineralize organic P in acidic soil (Chen Y. et al., 2020). But the unobserved correlation between a given bacterial taxa and organic P can also be due to the lack of P-related functional gene amplicon sequencing. This finding suggests that fungi are involved with organic P mineralization in maize system as well, however, the underlying mechanisms were not very clear and deserve further analysis.

### Optimizing P Fertilization Management for Coordination of Soil Microbial Community and Function

The structure and function of microbial communities are important to soil health. The effects of P gradient fertilization on soil P forms, microbial community, keystone taxa, and functional genes related to P cycling are important to understand the relationship among them. It was clear that low P rates (0 and 12.5 kg ha^–1^) resulted in higher fungal diversity but lower *phoD* gene copy numbers. Although the number of keystone taxa in the low P treatment was higher than that with the moderate and high P treatments, it was likely that competitive interaction between microbes was stronger, which might indicate that a greater number of keystone taxa is not necessarily correlated with healthy soil. At the same time, excess P (100 and 200 kg ha^–1^) decreased the fungal diversity, *phoD* gene copy numbers, and the number of the keystone taxa, which demonstrates that overuse of P fertilizer likely damaged the community and function of the microbiome. In comparison, the agronomically optimized P rates (25 to 50 kg ha^–1^) had specific community composition and keystone taxa, which were matched with optimal yield. In addition, the previous study conducted in the same field showed that the rhizosphere effects such as root morphology, mycorrhizal root colonization, etc. were improved around optimal P rates (Teng et al., 2013; Deng Y. et al., 2014; Deng et al., 2017). Our results showed that the gene copy numbers of functional genes such as *phoD* were significantly higher in the optimized P fertilization treatment than the other P fertilization treatments (Figure 6A) and was positively correlated with organic P (Figure 6B), demonstrating that the increase of organic P promoted the expression of the functional gene.

To our knowledge, this field study is the first to assess the systematic effects of long-term P application on alpha- and beta-diversity, keystone taxa, and functional genes linked to soil P forms in intensive maize cropping system. The findings of this study underscore the importance of the appropriate P fertilizer management in maize system for soil health.

Deeper research on the diversity of organic P speciation (measured by nuclear magnetic resonance) (Cade-Menun and Liu, 2014) and explore the relationships between organic P speciations and P mineralizing microbes (measured by metageome or metatrascriptome) (Liu J. et al., 2018), which could further unveil underlying microbial co-occurrence patters. To further identify the keystone taxa function and make good use of them such as synthetic microbial community technique (Niu et al., 2017), which may improve plant P uptake according to microbial method in order to decrease the amount of the fertilization.

## Conclusion

In summary, our study reveals that long-term P fertilization significantly decreased fungal but not bacterial alpha-diversity in a calcareous maize field. Community composition of bacteria and fungi at the phylum and class levels did not obviously shift, while composition significantly varied at the order and family levels under different rates of P application. The relative abundance of bacteria of *Anaerolineaceae*, *Sphingomonadaceae* and *Nitrosomonadaceae* were negatively correlated while bacteria of *Xanthomonadaceae*, *Nocardioidaceae* and *Micromonosporaceae* and fungi of *Chaetomiaceae* and *Cucurbitariaceae* were positively correlated with soil available P (Olsen P), inorganic P and total P concentration. The keystone taxa were specific at different P rates, and the number of keystone taxa decreased as P fertilization increased. One of the keystone taxa named BacOTU3771 belonging to Xanthomonadales was positively correlated with potential functional genes encoding enzymes such as glycerophosphoryl diester phosphodiesterase, acid phosphatase and positively correlated with guinoprotein glucose dehydrogenase The gene copy numbers of the *phoD* were positively correlated with organic P, and reached the highest level at the moderate P application rate. Our results revealed the systematic effect of P gradient fertilization on P forms, the microbial community, keystone taxa, and functional genes associated with P cycling and highlight the potential of moderate rates of P fertilization to maintain them in order to effectively attain soil health.

## Data Availability Statement

The datasets presented in this study can be found in online repositories. The names of the repository/repositories and accession number(s) can be found in the article/ Supplementary Material.

## Author Contributions

ML and XpC conceived the study. ML and WxZ contributed to the data analysis of bioinformatics. WxZ and XxC contributed to the soil sampling. ML contributed to draft the article. CZ, WZ, YD, FZ, PY, and XpC contributed to critically review and edit the manuscript. All authors contributed to the article and approved the submitted version.

## Conflict of Interest

The authors declare that the research was conducted in the absence of any commercial or financial relationships that could be construed as a potential conflict of interest.
