# Supplementary material for: Soil Microbial Composition and phoD Gene Abundance Are Sensitive to Phosphorus Level in a Long-Term Wheat-Maize Crop System
Source: Front Microbiol. 2021 Jan 14;11:605955. doi: 10.3389/fmicb.2020.605955 (PMC7873961; doi:10.3389/fmicb.2020.605955)
Supplement: Supplementary file 1 [file Data_Sheet_1.doc]

**Supplementary information**

**
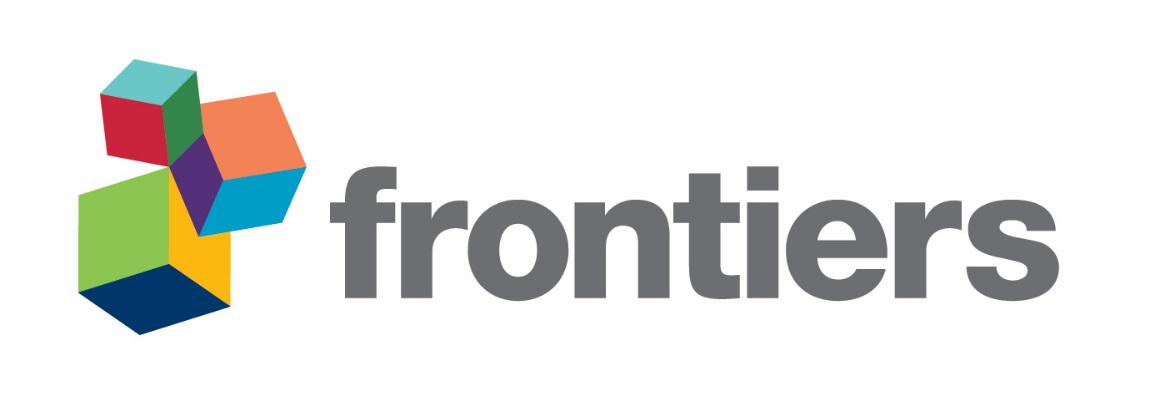
**

**Supplementary results**

We used heatmaps to illustrate relative enrichments in the bacterial and fungal compositions among P rate treatments at the family level (**Supplementary Figure S3**). The results show that the relative abundance of bacteria in *Anaeolineaceae*, *Sphingomonadaceae* and *Nitrosomonadaceae* decreased as P rates increased. As P fertilization increased, the relative abundance of the bacteria families *Micrococcaceae, Xanthomonadaceae*, *Nocardioidaceae*, *Micromonosporaceae* and the gene *MSB-1E8* increased, as did the relative abundance of the fungal family. While the relative abundance of *Rhodospirillaceae* and *Comamonadaceae* bacteria and *Cucurbitariaceae* fungi increased first and then decreased as P fertilization increased (**Supplementary Figure S3; Supplementary Table S4**).

**Supplementary figures and tables**

**Supplementary Figure S1.** Average annual maize grain yield from 2009 to 2013 (A) and from 2014 to 2018 (B) in response to P fertilization. Abbreviations: P0, P12.5, P25, P50, P100, P200 and P400 represent 0, 12.5, 25, 50, 100, 200 and 400 kg P ha^-1^, respectively. Different lowercase letters denote significantly different means at *P* < 0.05.


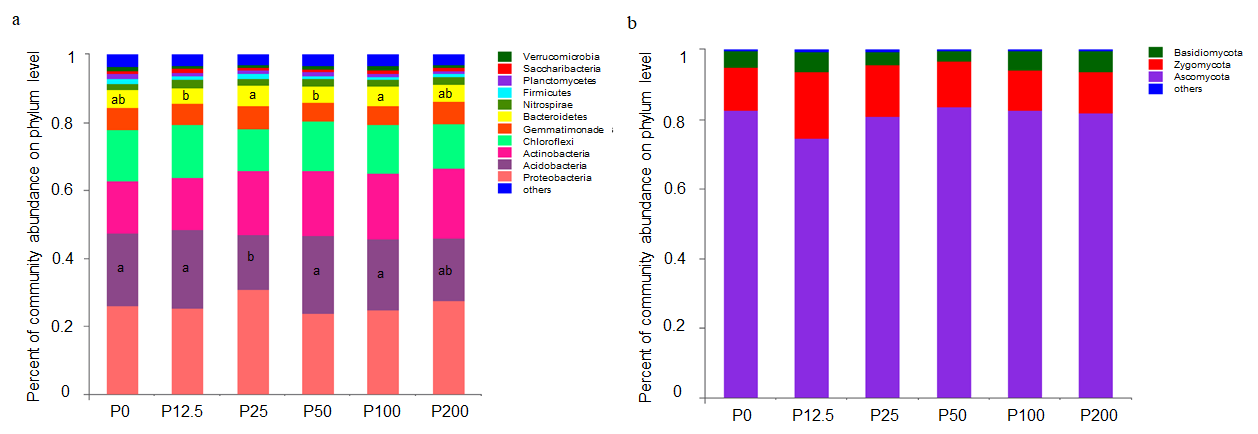


**Supplementary Figure S2.** Mean relative abundances of (a) bacterial and (b) fungal taxa at phylum in different long-term P fertilization treatments of the maize field. Different lowercase letters showed that there are significantly different at *P* < 0.05. Abbreviations: P0, P12.5, P25, P50, P100 and P200 represent 0, 12.5, 25, 50, 100 and 200 kg P ha^-1^, respectively.


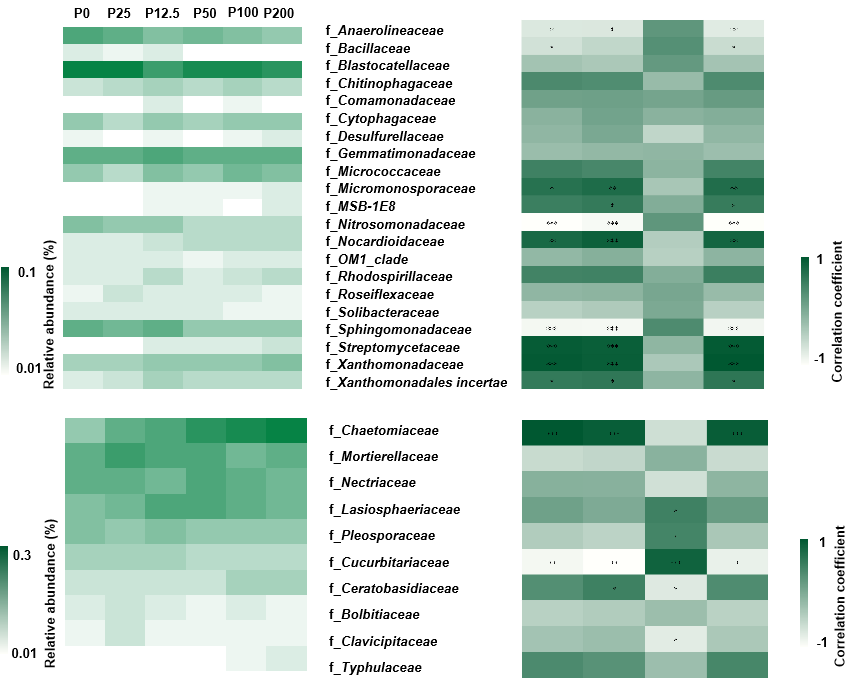


**Supplementary Figure S3.** Heatmaps of the dominant bacterial and fungal family. Taxa with relative abundance > 0.01% in at least one sample were included in the analysis. P0, P12.5, P25, P50, P100 and P200 represent 0, 12.5, 25, 50, 100 and 200 kg P ha^-1^, respectively.


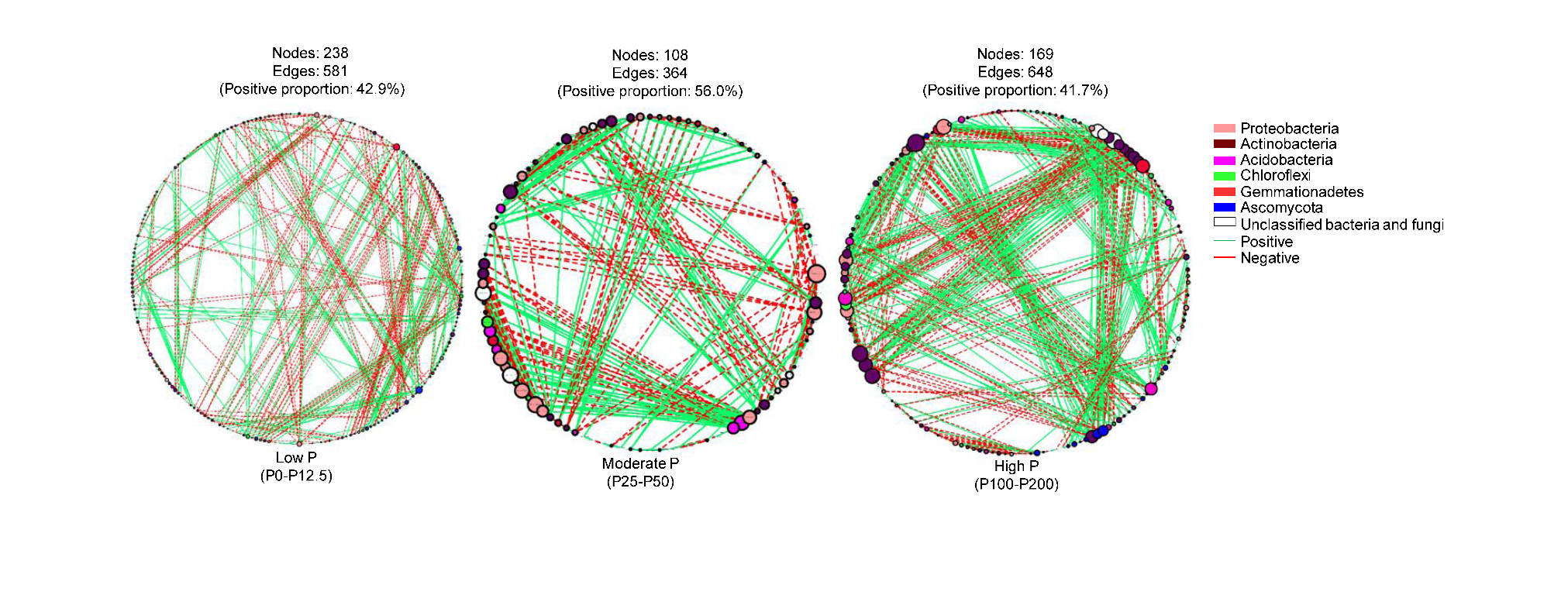


**Supplementary Figure S4.** Network of bacteria and fungi depending on P fertilization based on RMT (random matrix theory) analysis from OTU profiles. The size of each node is proportional to the number of connections. The color of nodes represents the taxa on the phylum classification. Solid lines represent a positive correlation, and dashed lines indicate a negative correlation. Abbreviations: P0, P12.5, P25, P50, P100 and P200 represent 0, 12.5, 25, 50, 100 and 200 kg P ha^-1^, respectively.

**Supplementary Table S1** Proportions of soil bacteria and fungi at the class level in different long-term P fertilization treatments of the maize field.

|  | Class | P0 | P12.5 | P25 | P50 | P100 | P200 |
| --- | --- | --- | --- | --- | --- | --- | --- |
|  | Acidobacteria | 21.41±2.01a | 23.11±6.01a | 16.11±3.01a | 22.81±3.51a | 20.81±2.11a | 18.21±5.31a |
|  | Actinobacteria | 15.41±1.71b | 15.41±1.31b | 18.81±1.71ab | 19.11±1.81a | 19.31±0.21a | 20.81±2.71a |
|  | Alphaproteobacteria | 12.31±0.81a | 12.01±2.11a | 14.81±1.71a | 10.91±1.11a | 11.41±0.81a | 12.51±2.01a |
|  | Gemmatimonadetes | 6.41±0.51a | 6.11±0.81a | 6.71±1.51a | 5.61±0.71a | 5.61±0.41a | 6.51±0.91a |
|  | Gammaproteobacteria | 5.21±0.41a | 5.21±0.91a | 6.51±1.01a | 5.61±0.51a | 6.11±0.11a | 7.01±0.81a |
|  | Betaproteobacteria | 5.21±0.51a | 4.61±0.71a | 5.21±1.51a | 3.71±0.51a | 3.81±0.21a | 3.81±0.31a |
|  | Deltaproteobacteria | 3.41±0.21a | 3.51±0.61a | 4.41±0.71a | 3.61±0.81a | 3.71±0.51a | 4.31±1.31a |
| Bacteria | Anaerolineae | 4.41±0.21a | 3.81±0.51a | 3.11±0.51a | 3.61±1.21a | 3.21±0.41a | 2.71±0.71a |
|  | Sphingobacteriia | 2.51±0.11a | 2.71±0.31a | 3.21±0.41a | 2.61±0.71a | 3.01±0.21a | 2.71±0.31a |
|  | Thermomicrobia | 2.51±0.11a | 2.51±0.41a | 2.31±0.51a | 2.61±0.01a | 2.81±0.21a | 2.41±0.41a |
|  | KD4-96 | 2.51±0.31a | 2.21±0.51a | 1.91±0.51a | 2.61±0.61a | 2.61±0.41a | 2.51±0.91a |
|  | Cytophagia | 2.71±0.21a | 1.81±0.41a | 2.81±0.61a | 2.11±0.11a | 2.51±0.31a | 2.31±0.41a |
|  | Nitrospira | 2.01±0.21a | 2.41±0.41a | 2.01±0.61a | 2.01±0.21a | 2.11±0.31a | 2.11±0.11a |
|  | Chloroflexia | 1.31±0.11a | 2.01±1.21a | 1.31±0.11a | 1.41±0.21a | 1.41±0.21a | 1.21±0.11a |
|  | Bacilli | 1.31±0.51a | 1.01±0.21a | 1.31±0.21a | 0.91±0.11a | 0.91±0.11a | 0.91±0.21a |
|  | Sordariomycetes | 63.81±4.01a | 58.21±58.21a | 64.71±7.51a | 70.31±2.51a | 70.91±4.71a | 69.71±5.31a |
|  | Dothideomycetes | 15.41±5.21a | 13.01±13.01a | 14.41±1.71a | 11.21±3.21a | 9.41±1.91a | 10.71±0.61a |
|  | Agaricomycetes | 4.31±2.81a | 5.01±5.01a | 3.41±2.21a | 2.71±2.31a | 5.41±2.51a | 5.91±2.31a |
|  | Eurotiomycetes | 1.01±0.31a | 0.91±0.91a | 0.61±0.11a | 1.11±0.91a | 1.01±0.21a | 0.81±0.81a |
| Fungi | Tremellomycetes | 0.61±0.31a | 0.61±0.61a | 0.41±0.11ab | 0.31±0.11b | 0.21±0.11b | 0.11±0.01b |
|  | Leotiomycetes | 0.21±0.01a | 0.91±0.91a | 0.21±0.11a | 0.21±0.11a | 0.21±0.21a | 0.21±0.11a |
|  | Chytridiomycetes | 0.21±0.11a | 0.51±0.51a | 0.31±0.11a | 0.31±0.11a | 0.21±0.01a | 0.21±0.21a |
|  | Pezizomycetes | 0.21±0.11a | 0.11±0.11a | 0.11±0.01a | 0.11±0.11a | 0.21±0.21a | 0.31±0.31a |
|  | Orbiliomycetes | 0.31±0.21a | 0.31±0.31a | 0.11±0.21a | 0.01±0.01a | 0.01±0.01a | 0.01±0.01a |
|  | Glomeromycetes | 0.01±0.01a | 0.11±0.11a | 0.11±0.11a | 0.11±0.01a | 0.01±0.01a | 0.01±0.01a |

Values are means ± standard error (n=3). Values in a column followed by different lowercase letters are significantly different at *P* < 0.05. Abbreviations: P0, P12.5, P25, P50, P100 and P200 represent 0, 12.5, 25, 50, 100 and 200 kg P ha^-1^, respectively.

**Supplementary Table S2** Pearson's correlation coefficients between soil properties and relative abundance in the class level of bacteria and fungi community

|  | Class | Olsen_P | Pi | Po | Pt |
| --- | --- | --- | --- | --- | --- |
|  | Acidobacteria | -0.148 | -0.189 | 0.195 | -0.131 |
|  | Actinobacteria | 0.769*** | 0.787*** | -0.329 | 0.742*** |
|  | Alphaproteobacteria | -0.205 | -0.164 | 0.137 | -0.162 |
|  | Gemmatimonadetes | -0.202 | -0.161 | -0.118 | -0.196 |
|  | Gammaproteobacteria | 0.598** | 0.598** | -0.271 | 0.620** |
|  | Betaproteobacteria | -0.776*** | -0.724*** | 0.235 | -0.745*** |
| Bacteria | Deltaproteobacteria | 0.162 | 0.230 | -0.195 | 0.146 |
|  | Anaerolineae | -0.589* | -0.585* | 0.201 | -0.620** |
|  | Sphingobacteriia | 0.163 | 0.131 | -0.046 | 0.131 |
|  | Thermomicrobia | 0.269 | 0.225 | -0.069 | 0.294 |
|  | KD4-96 | 0.201 | 0.209 | -0.077 | 0.259 |
|  | Cytophagia | -0.074 | 0.085 | -0.083 | -0.028 |
|  | Nitrospira | -0.018 | 0.084 | -0.277 | -0.015 |
|  | Chloroflexia | -0.318 | -0.307 | 0.072 | -0.361 |
|  | Bacilli | -0.596** | -0.514* | 0.312 | -0.569* |
|  | Sordariomycetes | 0.616** | 0.583* | -0.127 | 0.593** |
|  | Dothideomycetes | -0.608** | -0.699** | 0.684** | -0.554* |
|  | Agaricomycetes | 0.143 | 0.276 | -0.467 | 0.143 |
|  | Eurotiomycetes | -0.065 | -0.011 | -0.342 | -0.069 |
| Fungi | Tremellomycetes | -0.915*** | -0.915*** | 0.416 | -0.911*** |
|  | Leotiomycetes | -0.331 | -0.331 | -0.042 | -0.319 |
|  | Chytridiomycetes | -0.093 | -0.124 | 0.163 | -0.109 |
|  | Pezizomycetes | -0.013 | 0.038 | -0.381 | -0.024 |
|  | Orbiliomycetes | -0.847*** | -0.845*** | 0.386 | -0.832*** |
|  | Glomeromycetes | -0.275 | -0.331 | 0.639** | -0.237 |

**P* < 0.05, ***P* < 0.01，****P* < 0.001

**Supplementary Table S3** Results of envfit function showing the relationship of soil parameters with redundancy analysis of bacterial and fungal communities.

| Factor | Bacterial community | | Fungal community | |
| --- | --- | --- | --- | --- |
|  | R^2^ | *P* value | R^2^ | *P* value |
| Olsen P | 0.674 | 0.001 | 0.729 | 0.001 |
| Pi | 0.624 | 0.001 | 0.631 | 0.002 |
| Pt | 0.649 | 0.001 | 0.675 | 0.001 |
| Po | 0.179 | 0.220 | 0.546 | 0.003 |

The values showed the first principal component axis of plant community. *P* values are based on 999 permutations.

**Supplementary Table S4** Proportions of soil bacteria and fungi at the family level in different long-term P fertilization treatments of the maize field.

|  |  | Family | P0 | | P12.5 | P25 | P50 | P100 | P200 |
| --- | --- | --- | --- | --- | --- | --- | --- | --- | --- |
| Bacteria |  | Anaerolineaceae | | 0.044±0.001a | 0.038±0.003ab | 0.031±0.003b | 0.036±0.007ab | 0.032±0.002ab | 0.027±0.004b |
|  |  | Bacillaceae | | 0.011±0.002a | 0.008±0.001a | 0.011±0.002a | 0.007±0.000a | 0.007±0.000a | 0.008±0.001a |
|  |  | Blastocatellaceae | | 0.070±0.006ab | 0.072±0.008a | 0.051±0.009b | 0.067±0.007ab | 0.067±0.004ab | 0.055±0.011b |
|  |  | Chitinophagaceae | | 0.016±0.000a | 0.018±0.002a | 0.022±0.002a | 0.017±0.002a | 0.021±0.001a | 0.019±0.001a |
|  |  | Comamonadaceae | | 0.008±0.001ab | 0.007±0.000b | 0.010±0.002a | 0.007±0.001b | 0.009±0.000ab | 0.007±0.000ab |
|  |  | Cytophagaceae | | 0.027±0.001a | 0.018±0.002a | 0.028±0.004a | 0.021±0.001a | 0.025±0.002a | 0.023±0.002a |
|  |  | Desulfurellaceae | | 0.010±0.001a | 0.008±0.000a | 0.009±0.002a | 0.007±0.001a | 0.008±0.002a | 0.011±0.003a |
|  |  | Gemmatimonadaceae | | 0.041±0.002a | 0.041±0.005a | 0.047±0.006a | 0.037±0.003a | 0.038±0.002a | 0.042±0.004a |
|  |  | Micrococcaceae | | 0.026±0.005ab | 0.018±0.001b | 0.029±0.008ab | 0.028±0.000ab | 0.033±0.001a | 0.031±0.002a |
|  |  | Micromonosporaceae | | 0.008±0.001b | 0.007±0.001b | 0.009±0.002ab | 0.010±0.001ab | 0.010±0.001ab | 0.013±0.002a |
|  |  | MSB-1E8 | | 0.007±0.000b | 0.007±0.001b | 0.009±0.000ab | 0.008±0.001ab | 0.008±0.000b | 0.010±0.002ab |
|  |  | Nitrosomonadaceae | | 0.029±0.003a | 0.026±0.002abc | 0.026±0.005ab | 0.019±0.002bc | 0.017±0.001c | 0.017±0.001c |
|  |  | Nocardioidaceae | | 0.012±0.001bc | 0.010±0.000c | 0.015±0.003abc | 0.017±0.001ab | 0.018±0.001a | 0.018±0.002a |
|  |  | OM1_clade | | 0.011±0.001a | 0.012±0.000a | 0.010±0.002a | 0.009±0.000a | 0.010±0.001a | 0.014±0.003a |
|  |  | Rhodospirillaceae | | 0.012±0.000c | 0.013±0.002bc | 0.018±0.001a | 0.014±0.001abc | 0.015±0.000abc | 0.017±0.002ab |
|  |  | Roseiflexaceae | | 0.010±0.001a | 0.015±0.005a | 0.010±0.001a | 0.011±0.001a | 0.011±0.001a | 0.009±0.001a |
|  |  | Solibacteraceae__Subgroup_3_ | | 0.011±0.001a | 0.010±0.001a | 0.012±0.002a | 0.010±0.001a | 0.010±0.001a | 0.0100±0.001a |
|  |  | Sphingomonadaceae | | 0.036±0.002a | 0.034±0.003ab | 0.038±0.006a | 0.024±0.003b | 0.024±0.001b | 0.025±0.002b |
|  |  | Streptomycetaceae | | 0.006±0.001b | 0.007±0.001b | 0.013±0.001a | 0.013±0.002a | 0.012±0.001a | 0.015±0.001a |
|  |  | Xanthomonadaceae | | 0.020±0.001c | 0.020±0.001c | 0.026±0.003b | 0.023±0.000bc | 0.027±0.001ab | 0.031±0.001a |
|  |  | Xanthomonadales_Incertae | | 0.014±0.001b | 0.016±0.002ab | 0.020±0.001a | 0.017±0.001ab | 0.018±0.001ab | 0.020±0.002a |
| Fungi |  | Chaetomiaceae | | 0.065±0.006c | 0.121±0.022bc | 0.147±0.025bc | 0.219±0.058ab | 0.259±0.023a | 0.299±0.041a |
|  |  | Mortierellaceae | | 0.124±0.012b | 0.190±0.013a | 0.149±0.028ab | 0.130±0.012ab | 0.106±0.025b | 0.116±0.019b |
|  |  | Nectriaceae | | 0.117±0.009a | 0.125±0.013a | 0.094±0.008a | 0.130±0.026a | 0.127±0.009a | 0.107±0.025a |
|  |  | Lasiosphaeriaceae | | 0.083±0.020a | 0.101±0.019a | 0.137±0.017a | 0.134±0.04a | 0.112±0.027a | 0.105±0.010a |
|  |  | Pleosporaceae | | 0.075±0.013a | 0.061±0.004a | 0.074±0.008a | 0.061±0.014a | 0.055±0.008a | 0.066±0.001a |
|  |  | Cucurbitariaceae | | 0.039±0.007ab | 0.034±0.005abc | 0.047±0.003a | 0.029±0.004bc | 0.024±0.001c | 0.025±0.004bc |
|  |  | Ceratobasidiaceae | | 0.018±0.006a | 0.018±0.002a | 0.02±0.007a | 0.019±0.010a | 0.041±0.013a | 0.038±0.018a |
|  |  | Bolbitiaceae | | 0.005±0.003a | 0.013±0.009a | 0.005±0.004a | 0.002±0.001a | 0.006±0.001a | 0.002±0.001a |
|  |  | Clavicipitaceae | | 0.003±0.000a | 0.013±0.011a | 0.002±0.001a | 0.002±0.001a | 0.003±0.001a | 0.003±0.001a |
|  |  | Typhulaceae | | 0.014±0.001a | 0.013±0.004a | 0.012±0.007a | 0.011±0.004a | 0.005±0.005a | 0.011±0.011a |

Values are means ± standard error (n=3). Values in a column followed by different lowercase letters are significantly different at *P* < 0.05. Abbreviations: P0, P12.5, P25, P50, P100 and P200 represent 0, 12.5, 25, 50, 100 and 200 kg P ha^-1^, respectively.

**Supplementary Table S5** The abundance of microbial genes coding for enzymes involved in soil phosphorus mineralization and solubilization.

| KO | Phosphonatase (K05306) | Phytase (K01093) | Phosphotriesterase (K07048) | Glycerophosphoryl Diester Phosphodiesterase (K01126) | Alkaline Phosphatase (K01077) | Acid Phosphatase (K01078) | Acid Phosphatase (K09474) | Phosphate Inorganic Transporter (K03306) | Guinoprotein glucose dehydrogenase (K00117) |
| --- | --- | --- | --- | --- | --- | --- | --- | --- | --- |
| P0 | 310.9±33.3a | 189.7±6.8a | 1450.6±6.6a | 24084.2±295.5bc | 10941.3±842.9a | 663.8±4.3cd | 208.1±18.6a | 13524±185.6a | 2165.1±59.8abc |
| P12.5 | 278.5±30.6a | 206.2±19.6a | 1443.2±126.7a | 23662.2±664.4c | 12034.6±2199.6a | 643.1±49.6d | 150.7±6.8a | 13129.7±439.3a | 1892.8±65.1c |
| P25 | 368.5±48.3a | 237.8±19.2a | 1781.3±56.8a | 26103.1±362.5ab | 8466.1±870.7a | 875.6±59.8ab | 194.4±33.9a | 13418.3±445.1a | 2385.6±137a |
| P50 | 297.7±16.8a | 198±15.2a | 1750.5±217.3a | 25628.7±981.4abc | 13023.2±1575.8a | 747.6±14.7bcd | 174.9±15a | 14026.6±60.3a | 2070.6±60.5bc |
| P100 | 287.3±5.6a | 230.3±12.2a | 1695.7±91a | 25826.3±127.3ab | 11315.9±833.4a | 800.7±6.1bc | 206±6.3a | 14054.8±146.1a | 2277±88.4ab |
| P200 | 340.3±19.1a | 226.5±12.8a | 1896.3±192.5a | 26426.2±841.3a | 10208.6±1508.9a | 959.9±79.6a | 193.8±21.2a | 13893.3±142.8a | 2202.4±115.5ab |

Values are means ± standard error (n=3). Values in a column followed by different lowercase letters are significantly different at *P* < 0.05. Abbreviations: P0, P12.5, P25, P50, P100 and P200 represent 0, 12.5, 25, 50, 100 and 200 kg P ha^-1^, respectively.

**Supplementary Table S6** Pearson's correlation coefficients between the relative abundance of the keystone taxa and the potential functional gene abundance

| Keystone taxa | Phosphonatase  (K05306) | Phytase  (K01093) | Phosphotriesterase  (K07048) | Glycerophosphoryl  diester phosphodiesterase  (K01126) | Alkaline  phosphatase  (K01077) | Acid  Phosphatase  (K01078) | Acid phosphatase  (K09474) | Phosphate  inorganic transporter  (K03306) | Guinoprotein  glucose  dehydrogenase  (K00117) |
| --- | --- | --- | --- | --- | --- | --- | --- | --- | --- |
| BacOTU2884 | 0.638** | 0.458 | 0.702** | 0.621** | 0.333 | 0.607** | 0.198 | -0.115 | -0.383 |
| BacOTU1772 | 0.733** | 0.418 | 0.347 | 0.223 | 0.311 | 0.478* | 0.266 | -0.549* | -0.553* |
| BacOTU716 | -0.272 | -0.623** | -0.606** | -0.690** | -0.330 | -0.697** | -0.063 | -0.345 | 0.194 |
| BacOTU2218 | -0.265 | -0.288 | 0.105 | 0.081 | -0.043 | 0.201 | -0.046 | 0.630** | 0.215 |
| BacOTU289 | 0.009 | -0.548* | -0.464 | -0.521* | -0.104 | -0.420 | 0.035 | -0.319 | 0.007 |
| BacOTU1712 | 0.616** | 0.148 | -0.076 | -0.070 | 0.401 | 0.056 | 0.365 | -0.556* | -0.448 |
| BacOTU1513 | -0.546* | -0.437 | -0.764** | -0.795** | -0.583* | -0.772** | -0.545* | -0.277 | 0.414 |
| BacOTU4524 | 0.496* | -0.059 | -0.133 | -0.292 | 0.035 | -0.024 | 0.220 | -0.678** | -0.298 |
| BacOTU1200 | -0.361 | -0.459 | -0.356 | -0.482* | -0.455 | -0.458 | -0.307 | -0.410 | 0.019 |
| BacOTU3771 | 0.773** | 0.630** | 0.631** | 0.635** | 0.625** | 0.653** | 0.451 | -0.253 | -0.751** |
| BacOTU4750 | -0.532* | -0.278 | -0.235 | -0.090 | -0.236 | -0.194 | -0.373 | 0.705** | 0.670** |
| BacOTU759 | 0.014 | 0.398 | 0.054 | 0.163 | 0.168 | -0.002 | -0.121 | -0.107 | -0.143 |
| BacOTU1273 | 0.337 | -0.083 | -0.311 | -0.404 | 0.099 | -0.297 | 0.242 | -0.751** | -0.278 |
| BacOTU3197 | 0.521* | 0.089 | -0.167 | -0.256 | 0.164 | -0.167 | 0.195 | -0.738** | -0.319 |
| BacOTU3048 | 0.493* | 0.449 | 0.486* | 0.460 | 0.295 | 0.509* | 0.138 | -0.282 | -0.508* |

Values are means ± standard error (n=3). **P* < 0.05, ***P* < 0.01，****P* < 0.001
